# Porto-Pulmonary Hypertension and Hepato-Pulmonary Syndrome: Diagnostic Procedures and Therapeutic Management

**DOI:** 10.3390/diagnostics15141821

**Published:** 2025-07-19

**Authors:** Roberto G. Carbone, Francesco Puppo, Christopher A. Thomas, Vincenzo Savarino

**Affiliations:** 1 Department of Internal Medicine, University of Genoa, 16132 Genoa, Italy; puppof@unige.it (F.P.); vsavarin@unige.it (V.S.); 2 INOVA Fairfax Medical Center, Falls Church, VA 22042, USA; christopher.thomas@inova.org

**Keywords:** porto-pulmonary hypertension, hepato-pulmonary syndrome, pulmonary hypertension, cirrhosis

## Abstract

The common cause of porto-pulmonary hypertension and hepato-pulmonary syndrome is portal hypertension. Porto-pulmonary hypertension (PPHTN) is a form of pulmonary arterial hypertension, and hepato-pulmonary syndrome (HPS) occurs as a consequence of hepatic injury or vascular disorders. Demographic characteristics, pathophysiology, screening, differential diagnosis, and treatment of both disorders are treated in this review. Oxygen supply and other medical managements combined with vasodilator drugs are adopted for PPHTN and HPS treatment, but these two clinical conditions also represent an indication for liver transplantation. Despite poor evidence, PPHTN is treated as idiopathic pulmonary arterial hypertension. The latter is combined with improved pulmonary hemodynamics permitting lung transplant. Lung transplant improves PPHTN in one-half of patients and has been associated with longer survival in selected patients. However, the risk of the latter procedure can be relevant as it is closely related to PPHTN severity. Large clinical trials and international guidelines may have a predominant role in increasing our knowledge of both PPHNT and HPS and in improving their outcome by favoring an early diagnosis and more accurate treatment.

## 1. Introduction

The common cause of porto-pulmonary hypertension and hepato-pulmonary syndrome is portal hypertension [1]. Porto-pulmonary hypertension (PPHTN) refers to pulmonary arterial hypertension (PAH) that is associated with portal hypertension, affecting prognosis, medical treatment options, and liver transplant eligibility [2]. PPHTN is a recognized complication of portal hypertension due to chronic liver disease or extra-hepatic causes. Notably, approximately 10% of PPHTN patients have portal hypertension without cirrhosis. Cirrhosis has been found especially in Schistosoma Mansoni infection [3]. The patho-physiology of PPHTN remains poorly understood. It is detected in cirrhotic and non-cirrhotic portal hypertension and is not associated with the etiology of liver disease or the severity of portal hypertension [2]. Important risk factors are autoimmune diseases and female sex [4]. PPHTN belongs to the World Health Organization (WHO) group 1 PAH classification [5] and is diagnosed when PAH is detected in a patient who has co–existing portal hypertension and no other causes of PAH (e.g., connective tissue disease, congenital heart disease, human immunodeficiency virus infection, or stimulant use). Hepato-pulmonary syndrome (HPS) is characterized by gas exchange abnormalities due to pulmonary vaso-dilation and right-to-left-shunting [6]. The definitions of PPHTN and HPS are reported in Table 1.

## 2. Porto-Pulmonary Hypertension

Portal hypertension develops as a consequence of several causes, notably cirrhosis, schistosomiasis infection, or portal vein thrombosis, and is closely related to resistance to portal blood flow leading to comorbidities like variceal bleeding, ascites, hepatic encephalopathy, and porto-pulmonary hypertension (PPHTN). Portal hypertension is considered clinically significant when the hepatic venous pressure gradient is greater than 10 mmHg [7].

PPHTN has the same hemodynamic definition as other PAH subgroups [5]:

- Mean pulmonary artery pressure (mPAP) > 20 mmHg
- Pulmonary capillary wedge pressure (PCWP) < 15 mmHg
- Pulmonary vascular resistance (PVR) ≥ 2 Wood units (WU)

The 2022 European Society of Cardiology (ESC)/European Respiratory Society (ERS) lowered the PVR cutoff for PAH from 3 to 2 WU; however, it is still unclear how this change will affect diagnosis and management of patients with PPHTN.

### 2.1. Demographics and Characteristics of Patients

The prevalence of PPHTN is difficult to estimate. McDonnel et al. [8] reported that patients with cirrhosis have a prevalence of PAH equal to 0.73%. In the United States and Europe, the prevalence of PAH ranges from 15 to 60 subjects per million, with PPHTN prevalence estimated at about 5–15% of PAH patients [9,10]. PPHTN incidence is closely associated with age and cirrhosis increase. Notably, in North America, the prevalence of cirrhosis increased 1.6- to twofold in the last two decades [11]. Humbert et al. [12] reported that PPHTN was the third most common form of idiopathic PAH in a population-based epidemiologic study in France. The French pulmonary hypertension registry reported that the PPHTN 1-, 3-, and 5-year survival rates were 84%, 69%, and 51%, respectively [12]. This data is analogous to the data reported for idiopathic PAH patients. By contrast, data from the United States based on the REVEAL registry showed that PPHTN patients have a worse survival with respect to PAH patients, i.e., 67% and 85% survival at 2 years, respectively [13].

### 2.2. Pathophysiology

**Figure 1:**
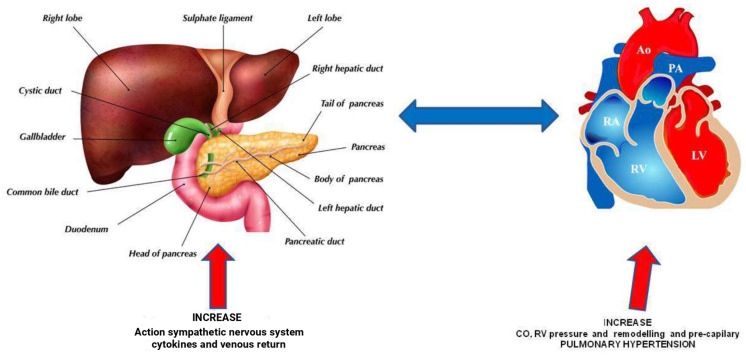
Interplay between liver circulation and right heart in pulmonary hypertension. RA: Right atrium, Ao: Aorta, PA: Pulmonary artery, LV: Left ventricle, RV: Right ventricle.

**Figure 2:**
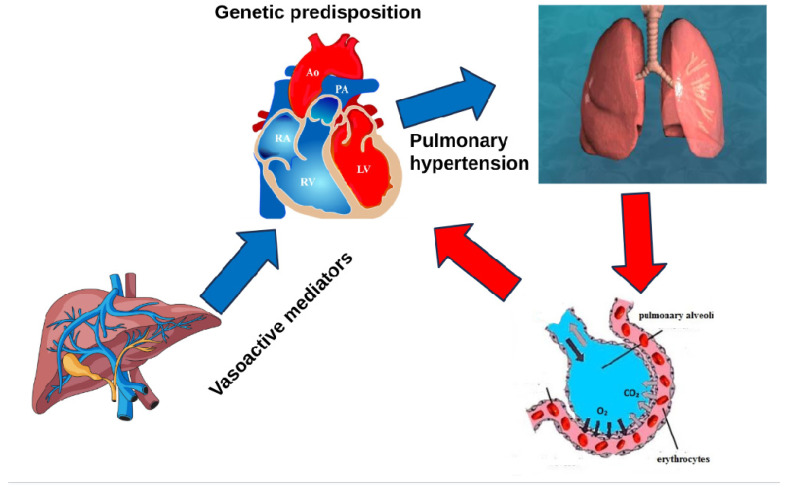
Genetic predisposition and imbalance between vasoconstrictive and vasodilator mediators in porto-pulmonary hypertension. RA: Right atrium, Ao: Aorta, PA: Pulmonary artery, LV: Left ventricle, RV: Right ventricle.

PPHTN develops through a complex set of mechanisms. Increased portal venous pressure leads to the opening of preexisting and new porto-systemic collaterals and to an increase in splanchnic blood flow [14,15]. This leads to a decrease in systemic vascular resistance with consequent hyperdynamic circulation. As blood begins to bypass the liver and progress directly to pulmonary vascular bed and systemic circulation, an imbalance between vasoconstrictive and vasodilator mediators (including nitric oxide, prostacyclin, endothelin, and serotonin) and endothelial growth factor occurs [16] (Figure 1 and Figure 2). Additionally, pulmonary vascular responses triggered by lipopolysaccharide exposure from gut bacterial translocation may trigger an exaggerated inflammation in susceptible individuals with bone morphogenic protein receptor type 2 (BMPR2) deficiency [17]. Furthermore, some findings suggest that single nucleotide polymorphisms in the aromatase gene (CYP19A1) leading to elevated estrogens levels likely play a role in PPHTN pathogenesis [18].

All these underlying mechanisms result in three major damages to the pulmonary vascular bed: (i) endothelial proliferation leading to an increase in wall thickness and pulmonary artery constriction; (ii) smooth muscle proliferation with consequent medial wall increase and development of plexiform lesions; and (iii) platelet aggregation with development of thrombosis [19]. These changes to pulmonary arterioles are akin to changes seen in PAH related to other causes. More research is needed to further clarify the multifactorial mechanisms that result in PPHTN.

### 2.3. New Insights in Pathophysiology

**Figure 3:**
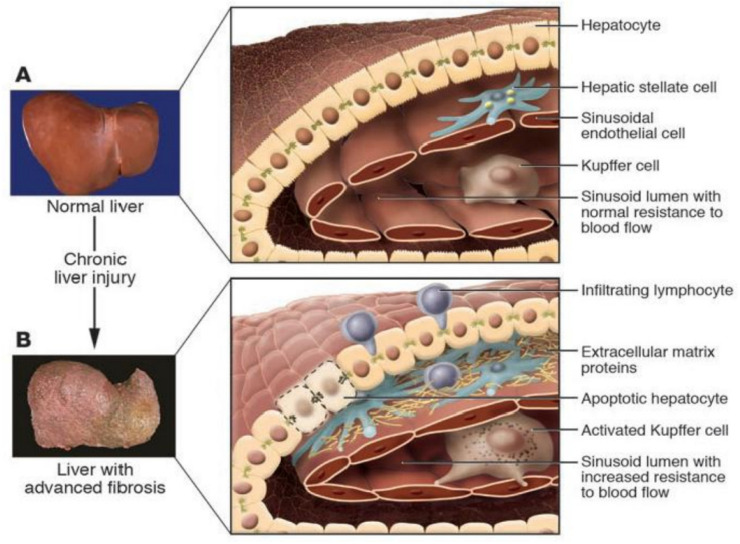
(**A**) In a healthy liver, hepatic stellate cells (HSC) or lipocytes are involved in the turnover of the extracellular matrix. (**B**) Upon liver injury, HSCs transition from a quiescent phenotype to a myofibroblast-like phenotype, passing through an intermediate stage called transitional cells. These changes are reversible, but repair of liver tissue damage leads to recurrence and activation of HSCs.

Interestingly, in the study of PPHTN pathogenesis, hepatic stellate cells (HSCs), also known as Ito cells or lipocytes, play a key role in liver fibrosis and cirrhosis. Notably, exploring the liver function, the HSCs are essential in various processes, such as homeostasis, extracellular matrix maintenance, repair, and regeneration. Laboratory research provides high-quality primary HSCs to support cutting-edge research into liver disease pathogenesis and therapeutic development. In a healthy liver, HSCs maintain extracellular matrix integrity, regulate microcirculation, and store vitamin A. However, following liver injury, these cells undergo activation, transforming themselves into myofibroblast-like cells and becoming the primary source of type I collagen in fibrotic tissue. Activated HSCs contribute to hepatic inflammation, fibrosis, and intrahepatic portal hypertension, making them a critical focus for studies on liver disease progression and treatment. A recent study highlights the heterogeneity of activated HSCs, revealing distinct populations with proliferative, inflammatory, or fibrogenic properties. Understanding the molecular regulation of HSC activation is key to developing novel therapeutic strategies for hepatic fibrosis, potentially reducing morbidity and mortality in patients with chronic liver conditions [20,21] (Figure 3).

### 2.4. Screening and Diagnosis

In general, PPHTN symptoms are represented by dyspnea, exercise intolerance, chest pain, syncope, or near syncope. On clinical examinations, patients may show signs suggesting right heart failure, such as peripheral edema, ascites, and alteration of heart sounds that are wide and split and suggest tricuspid regurgitation.

**Figure 4:**
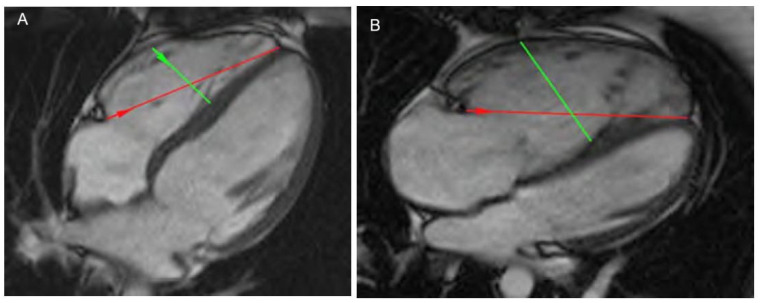
(**A**) Computed tomography scan of normal heart. (**B**) Computed tomography scan in porto-pulmonary hypertension showing cardiomegaly with right heart enlargement. Green arrows: Mid-Cavity diameter; Red arrows: Longitudinal diameter.

Chest x-ray may show cardiomegaly with right heart enlargement that is confirmed by computed tomography scan (Figure 4). Pulmonary function tests may show a reduction in diffusing capacity for carbon monoxide (DLCO) and impaired six-minute walk distance (6MWD), revealing lower exercise tolerance. Electrocardiograms may suggest right atrial enlargement, right ventricular hypertrophy, right axis deviation, and right bundle branch block [22]. The laboratory should include serological screening tests and pulmonary function tests with lung volumes, DLco evaluation, and 6MWD to exclude other causes of PAH [23]. Additionally, a ventilation/perfusion scan should be done to evaluate the presence of chronic thrombo-embolic pulmonary hypertension (especially in the study of portal vein thrombosis) [22,23].

When PPHTN is suspected, the best initial screening is performed by trans-thoracic echocardiogram (TTE). If the right ventricular systolic pressure (RVSP) assessed by TTE is ≥50 mmHg, right heart catheterization (RHC) is required to confirm this value. For a definite PPHTN diagnosis, PAH must be confirmed by the following hemodynamic parameters: (i) mPAP > 25 mmHg; (ii) PAWP < 15 mmHg; (iii) PVR > 240 dyn/s/cm^−5^ (3 Wood units). The 6th World Symposium on Pulmonary Hypertension updated the hemodynamic definition of PH as mPAP > 20 mmHg. Portal hypertension is defined by portal venous gradient > 5 mmHg [24].

The frequency of screening echocardiography is unknown; therefore, it is reasonable to perform annual echocardiography in patients awaiting liver transplantation [25].

In this context, Krowka et al. [6] identified a moderate correlation between right ventricular systolic pressure and pulmonary arterial systolic pressure measured on RHC. Prognosis of non-treated patients is 2–5 yrs and is poor in comparison with patients with idiopathic PAH [6]. However, treatment of pulmonary arterial hypertension may lead to improvement of pulmonary hemodynamics while waiting for liver transplantation.

**Figure 5:**
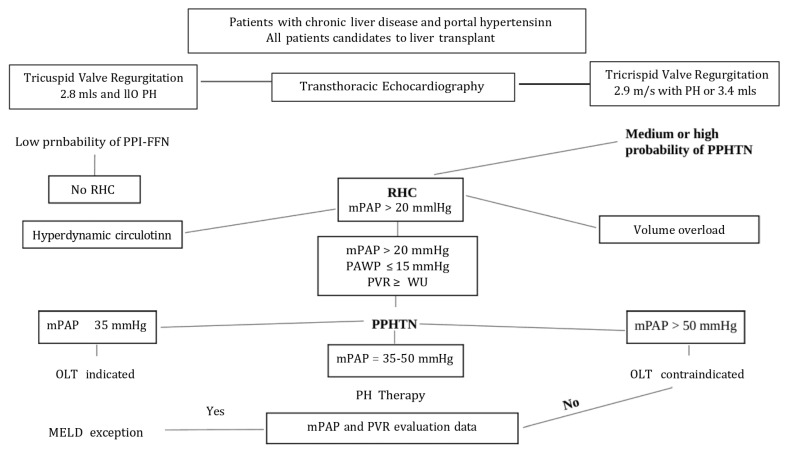
Algorithm of patients’ candidacy for liver transplantation (adapted from Jasso-Baltazar et al. [26]).

Furthermore, Jasso–Baltazar et al. [26] proposed an algorithm suggesting current clinical criteria for accurate selection of patients’ candidacy for liver transplantation. We report a modified revision of this algorithm (Figure 5). Patients with advanced liver disease waiting for transplantation should be screened for porto-pulmonary hypertension with echocardiography annually [5]. However, the procedure is controversial; a lot of centers skip TTE and do serial RHC, since the presence of PPHTN confers so much risk for pre-operative mortality.

### 2.5. Treatment

PPHTN leads to right heart insufficiency and death. The goal of treatment is to obtain partial regression of symptoms, improvement of pulmonary functional capacity and quality of life, and decrease of mortality.

Nevertheless, only limited randomized controlled trials have been performed, and the evidence that PAH treatments are effective in PPHTN derives from observational studies. Furthermore, the meta-analysis of 12 studies showed that PAH therapy was associated with an improvement in pulmonary hemodynamics. This finding has been recently confirmed by a meta-analysis including 26 studies and over 1000 PPHTN patients [25].

Currently, PPHTN treatments comprise four major drug classes: (1) prostacyclin and their analogs causing vasodilation, (2) phosphodiesterase 5 inhibitors (like sildenafil) blocking apoptosis, (3) endothelin receptor antagonists interfering with endothelin A and B receptor pathways, and (4) guanylate cyclase stimulators increasing NO function.

In detail, prostacyclin is a vasodilator with an antiplatelet aggregation effect promoting visceral vasodilation and blocking vascular proliferation. Prostacyclin analogues regroup epoprostenol, treprostinil, and iloprost, which improve pulmonary hemodynamic and survival outcome in PPHTN patients. Sildenafil is a pulmonary vasoactive drug with efficacy in PAH treatment. PPHTN patients respond well to sildenafil monotherapy and in association with iloprost.

Endothelin receptor antagonists such as bosentan, macitentan, and ambrisentan are further drug options for PAH treatment. Bosentan, a non-selective endothelin A and B receptor antagonist, reduces PVR, is well tolerated, and improves PPHTN patients’ outcomes. Riociguat, a guanylate cyclase stimulator approved for PAH treatment, increases NO and induces pulmonary vasodilation. PPHTN patients treated with riociguat showed excellent improvements in 6-MWD and WHO Functional Class and significant PVR reduction at 12 weeks.

PAH vasodilator treatment associated with liver transplantation demonstrated outcome improvement. Of note, patients who did not undergo liver transplant (LT) were affected by several comorbidities [27].

In agreement with the International Liver Transplant Society Practice Guidelines, mPAP ≥ 45 mmHg is considered an absolute contraindication for LT. Based on these guidelines, PPHTN patients without criteria for LT must be treated with PAH therapy. The Spanish PAH Registry demonstrated that patients with PPHTN treated with PAH therapy showed higher survival with respect to untreated patients. Furthermore, overall survival rates at 1, 3, and 5 years were 81.1%, 66.4%, and 49.8% in the treated group in comparison with 53.3%, 35.5%, and 17.8%, in the untreated group (*p* < 0.001) [26]. Notably, Savale et al. and Humbert et al. [10,12] evaluated the effectiveness of PAH-vasodilator treatment in PPHTN patients included in the French Pulmonary Hypertension Registry. In summary, 57% of 637 patients analyzed were affected by mild cirrhosis, and PAH vasodilator therapy was performed in 74% of patients. Deroo et al. [28] demonstrated in their meta-analysis that pulmonary hemodynamics and survival were significantly better in PPHTN patients treated with vasodilators, liver transplantation, or both than in patients treated with vasodilators alone.

Recent studies confirm the benefit of liver transplants on the survival of PPHTN patients treated with vasodilator therapy. A pooled analysis of the clinical outcomes of patients from Mayo Clinic liver transplantation centers showed that 50 out of 228 patients treated with PA -specific therapy and undergoing liver transplantation achieved significant hemodynamic improvement. Notably, a significant number of patients (42%) were able to discontinue and remained off PAH-targeted therapy after LT [29,30].

Identifying the beneficial effects of PAH-specific therapy in combination with liver transplantation is important because the transplantation procedure is not without complications. For example, during liver graft reperfusion, severe systemic hemodynamic changes, such as cardiac output increase, are often observed, which can exacerbate PH and cause potential right-sided heart failure with liver graft congestion and reverse flow in the hepatic veins. This condition is extremely difficult to treat with existing drugs, such as milrinone, nitric oxide, and norepinephrine [29].

Overall, PPHTN patients not undergoing LT and medical therapy experience poor outcomes, with a 5-year survival rate of 14% [10], whereas patients treated with vasodilators combined with liver transplant have a better prognosis than those receiving vasodilators treatment alone or not receiving treatment [10]. Prompt identification of selected LT candidates is essential to optimize surgical planning and reduce perioperative mortality risk [31].

The first therapeutic approach includes oxygen support therapy for hypoxemia (resting, walking, and nocturnal) in combination with physiotherapy. Additionally, pulmonary vasodilator therapy may be introduced more to reduce pulmonary vascular resistance than to decrease mPAP. In the PORTICO randomized clinical trial, patients treated with macitentan showed a 37% decrease in pulmonary vascular resistance, but only a 14% mPAP drop and a 19% cardiac index increase were observed [32]. A limitation of the PORTICO trial is the exclusion of patients with Child–Pugh class C liver disease and patients with MELD score > 19, which are groups with poor survival.

The Child–Pugh (CPS) classification reports an empiric system including five variables, bilirubin, albumin, prothrombin time, ascites, and encephalopathy (Table 2). CPS subdivides patients into low (class A), intermediate (class B), and poor (class C) risk, based on advanced liver disease, and is predictive of outcome. However, CPS classification, being a subjective variability evaluation of parameters, led to the development of a” model for end stage liver disease” (MELD) score based on laboratory tests only, more accurate and objective with respect to CPS [33].

Prospective cohort data from the French Pulmonary Hypertension Registry demonstrated that patients with PPHTN who started pulmonary vasodilators have significant improvements in New York Heart Association (NYHA) functional class, 6-MWD, and PVR [12].

A meta-analysis of 26 non-randomized and randomized studies, including a large sample of 1019 patients, confirmed that pulmonary hypertension therapies in patients with PPHTN improved pulmonary hemodynamics, and 44% of patients treated with pulmonary vasodilators became eligible for liver transplantation [28].

Observational studies suggest that PAH treatment ameliorates prognosis and eligibility, reducing risks related to liver transplantation, whereas it is not still known if PAH treatment improves transplant free survival, because potential survival advantage is curtailed by advanced liver disease and comorbidities [34]. Tokushige et al. [24] showed that PAH-specific therapeutic approaches with endothelin receptor agonists, phosphodiesterase-5 inhibitors, or prostacyclin improve the survival rate of PPHTN patients. However, long-term effects of these drugs and their influence on PPHTN prognosis remain unclear. Krowka et al. [35] evaluated 43 patients with PPHTN assessed by RHC who subsequently underwent liver transplantation. Patients were divided into three groups based on the severity of PPHTN. The first group had mild pulmonary hypertension (mPAP between 25 and 35 mmHg), the second group had moderate pulmonary hypertension (mPAP between 35 and 50 mmHg), and the third group had severe pulmonary hypertension (mPAP greater than 50 mmHg). Mortality was 100% and 50% in patients with severe and moderate PPHTN, respectively. Most deaths occurred after liver transplant. No mortality was reported among patients with mild PPHTN. After this study, it was agreed that patients with PPHTN and higher mPAP were at high risk of death, and therefore pre-transplant PPHTN treatment would be required, although studies in this area are limited and specifically targeted to the treatment of idiopathic pulmonary hypertension (IPAH) [35].

Interestingly, Giannini et al. [36] studied the role of clinical portal hypertension after hepatic resection for hepatocellular carcinoma in 152 patients with compensate cirrhosis and concluded that portal hypertension has no influence on patients’ survival with well-compensated cirrhosis undergoing hepatic resection for hepatocellular carcinoma.

Calcium channel blockers are not used in patients with porto-pulmonary hypertension because they can worsen systemic hypotension and increase portal hypertension. Prostacyclin agonists, endothelin receptor agonists, phosphodiesterase inhibitors, and guanylate cyclase stimulants are recommended with a high level of evidence for patients in NYHA classes II and III and with a low level of evidence for NYHA class IV patients [37]. The unique role of PAH treatment in the latter PPHTN patients’ group is to favor liver transplantation. Interestingly, sildenafil was found to improve NYHA class, exercise tolerance, and hemodynamics in PPHTN patients.

### 2.6. Liver Transplant

Since 2006, the Model for End-stage Liver Disease (MELD) score is utilized to select LT candidates with porto-pulmonary hypertension with the following priority criteria: (i) PPHTN diagnosed on RHC, (ii) PAH and initiation of treatment with hemodynamic improvement (mPAP, 35 mmHg, PVR, 5WU), and (iii) MELD exception, indicating priority to receive transplant of patients with MELD score not reflecting urgency, evaluated every 3 months. MELD system has several limitations. First, an important number of PPHTN patients lack pre- and post-transplant hemodynamic parameters (for example CO, cardiac index), which are useful to predict the risk of cardiopulmonary complications. Second, favorable significative data of post-transplant outcomes generated from small case series with possible bias lead falsely to consider PPHTN as a substantial and definitive transplant indication. Evaluating 155 LT waitlist candidates’ overall mortality showed that it was higher in patients granted PPHTN MELD exception points in comparison with all non-exception waitlist candidates.

Notably, in a cohort of 190 patients approved to receive a MELD score, initial PVR and liver severity were predictive of waitlist mortality. Data analysis suggests that the PPHTN MELD score requires additional variables (for example, baseline PVR and MELD score) for an accurate pre- and postoperative risk for transplant candidates [31].

### 2.7. Outcome

In the era of the AASLD/ILTS guidelines and MELD, data on a PPHTN course after LT are yet scarce. In one retrospective cohort study, hemodynamics and survival at 6 months, 1 year, and 3 years, were 80%, 77%, and 77%, respectively, for 35 patients with PPHTN. In the study, 27/35 patients survived more than 6 months after LT, and all were able to be weaned from intravenous epoprostenol. Notably, most patients improved their hemodynamics after LT showed PAP < 25 mmHg at the last follow-up. The data reported suggest performing the LT procedure only in selected candidates. Summarizing, the outcome of PPHTN is poor and closely related to severity of pulmonary hypertension and liver disease [24]. Survival after liver transplantation in porto-pulmonary hypertension is reported in Table 3.

A single published meta-analysis [29] evaluating 11 trials involving 37,686 transplant recipients showed that PPHTN patients had significantly increased 1-year mortality compared to the control group, while graft loss and 30-day mortality were similar.

## 3. Hepato-Pulmonary Syndrome

Hepato-pulmonary syndrome (HPS) is characterized by gas exchange abnormalities due to pulmonary vasodilation and right-to-left-shunting. This contrasts with PPHTN, which is characterized by PH secondary to increased pulmonary vascular resistance leading to right ventricular failure. HPS is a disorder generally seen in patients with portal hypertension, with or without cirrhosis. More rarely, it can occur in patients with vascular abnormalities that alter liver-lung blood flow, as well as in acute or chronic hepatitis or acute liver failure. It is characterized by impaired gas exchange due to a deficiency in the pulmonary microvascular bed. There may also be a genetic predisposition [38]. HPS diagnosis is based on the diagnostic criteria reported in Table 1 [6].

### 3.1. Demographics and Characteristics of Patients

HPS incidence ranges from 5% to 32% in cirrhotic patients and occurs in 25% of patients suffering from chronic liver disease or PPHTN associated with cirrhosis. HPS severity is not correlated to the severity of liver disease [39]. HPS prevalence is estimated to range from 4% to 47% in patients with chronic liver disease [40]. The wide range of prevalence is attributable to the heterogeneity of diagnostic criteria and populations studied [41].

### 3.2. Pathophysiology

HPS patho-physiology is characterized by lung capillaries dilation determined by nitric oxide (NO) and endothelin 1 production. This process is favored by intestinal bacteria endotoxemia occurring in liver disease and leading to massive macrophages and monocytes infiltration in the lungs [42,43].

Monocytes produce tumor necrosis factor alpha (TNF α) within pulmonary vessels that activate NO synthetase. The latter, associated with bacteria accumulation, increases NO and heme oxygenase levels. Heme oxygenase decreases heme and increases carbon monoxide (CO) levels. Then, NO and CO develop pulmonary vasodilation. Additionally, TNF α in combination with macrophages and monocytes activate vascular endothelial growth factor (VEGF), leading to angiogenesis in pulmonary vascular tissue. Angiogenesis and vasodilation lead to arterial-venous (AV) shunt development, the so-called shunt effect, and cause ventilation/perfusion (VA/Q) mismatch in the inferior lobes of both lungs. AV shunts and alteration of VA/Q determine two different types of vascular pulmonary vasodilation: type I that involves pre-capillary arterial/alveolar gradient developing hypoxemia and type II that involves vasodilation larger vessels. The consequence of this process is defined as chronic respiratory failure with permanent brain and heart injury that, if untreated, can be fatal.

### 3.3. Screening and Diagnosis

HPS diagnostic criteria include the triad: (1) presence of liver disease and portal hypertension; (2) room air/alveolar-arterial oxygen gradient [P (A-a) O_2_ gradient] > 15 mmHg in subjects < 65 years or >20 mmHg in subjects ≥ 65 years; and (3) evidence of intrapulmonary vascular dilation in lung basal lobes [42,43]. Another criterion for HPS diagnosis is to estimate portal hypertension degree with and without cirrhosis. HPS severity is defined on partial pressure of arterial blood oxygen (PaO_2_) levels: (i) mild ≥ 80 mmHg; (ii) moderate ≥ 60 mmHg to <80 mmHg; (iii) severe ≥ 60 mmHg to <50 mmHg; (iv) very severe < 50 mmHg.

The most common HPS form is associated with chronic liver disease and/or cirrhosis, although the severity of liver disease is not correlated with HPS increase. HPS has an insidious onset, and early-stage patients are asymptomatic or suffer from dyspnea, which is a common symptom in chronic liver disease. HPS may occur in combination with cardio-pulmonary disease, which can determine V/Q abnormalities.

The physical examination shows the following: (1) cyanosis; (2) digital clubbing; (3) diffuse telangiectasia; (4) platypnoea (worsening dyspnea related to the cline and upright position); and (5) orthodeoxia (decrease in PaO_2_ of about 5 mmHg during the changes of body position).

The first clinical screening is to evaluate a possible decrease in blood oxygen saturation utilizing a pulse oximeter. If desaturation is found, the patient must undergo arterial blood gas analysis to accurately define PaO_2_ and A-aO_2_ parameters.

The gold standard HPS diagnostic procedure is TTE associated with intravenous saline solution injection. Normally, micro-bubbles follow pulmonary vascular circulation to perform gas exchange into the alveoli, and vice versa in HPS vasodilation of the pulmonary arteries, and A-V shunts allow micro-bubbles to jump in the left atrium and ventriculum. This process is detected by TTE showing pulmonary vasodilation. Trans-esophageal echocardiogram is more accurate than TTE. However, several complications can occur during this procedure caused by esophageal varices present in many cirrhotic patients [44].

Radioactive lung perfusion scanning is another text to evaluate pulmonary vasodilation. This procedure does not have the possibility to estimate pulmonary/cardiac O_2_ exchange, whereas it has the capacity to identify hypoxemia in patients with concomitant pulmonary vascular disease. Pulmonary angiography can be useful to distinguish type I and type II HPS. Limitations of this procedure include its high cost, invasiveness, and lower sensibility in comparison with TTE.

Complementary diagnostic tests are as follows: (1) chest-x ray that could show opacities concomitant with pulmonary vasodilation and exclude other pulmonary diseases; (2) CT scan to better define above findings; and (3) pulmonary function tests that can reveal DLCO decrease.

### 3.4. Treatment

Oxygen therapy is recommended for patients with severe hypoxemia waiting for liver transplantation to improve exercise, tolerance, and quality of life. Liver transplantation is the only treatment that improves HPS survival and prognosis and that can reduce hypoxia for at least 6–12 months.

Other recommended medical treatments include pentoxifylline, mycophenolate mofetil, aspirin, nitric oxide, and somatostatin [45].

#### 3.4.1. Endothelin Receptor Antagonists

Endothelin receptor antagonists (ERAs) including bosentan, macitentan, and ambrisentan are used in PAH patients to improve both hemodynamic and clinical parameters. ERAs have been increasingly used in the PPHTN population. However, ERAs may adversely affect liver function and should be used with caution in patients with moderate to severe hepatic impairment. Some studies have shown that PPHTN patients treated with bosentan have clinical and hemodynamic improvements, contributing to increased overall survival, although with a small risk of hepatological side effects [46,47,48]. Two clinical trials have identified the efficacy of macitentan and ambrisentan in PPHTN. Interestingly, the randomized, multicenter, double-blind, placebo-controlled PORTICO trial evaluated the efficacy of macitentan in PPHTN patients. It demonstrated that the drug produced hemodynamic benefit, reducing PVR by 35% both in treatment-naive patients and in patients on background PDE-5i therapy, with minimal concerns about hepatic side effects. Additionally, a small, multicenter, uncontrolled, open-label study reported that ambrisentan monotherapy improved hemodynamics and functional class in 31 PPHTN patients [33,49].

#### 3.4.2. Phosphodiesterase-5 Inhibitors

PDE-5 inhibitors (PDE-5i) enhance pulmonary vasodilation using the nitric oxide (NO) pathway by inhibiting the degradation of cyclic guanosine monophosphate. Although large clinical trials are lacking, results from smaller studies suggest that both sildenafil and tadalafil can be safely administered to patients with PPHTN [50]. With regard to PDE-5i efficacy, small uncontrolled studies have demonstrated that sildenafil treatment of PPHTN patients is closely related to reduction in PVR and mPAP, increase in CO, and overall improvements in exercise capacity and right ventricular (RV) function [50,51,52,53,54].

#### 3.4.3. Soluble Guanylate Cyclase Stimulator

Safety and efficacy of riociguat, a soluble guanylate cyclase (sGC) stimulator, were evaluated in 11 PPHTN patients enrolled in PATENT-1 and PATENT-2 clinical trials. Although definitive conclusions cannot be drawn from such a small subset of patients, riociguat may represent a therapeutic option for these individuals [30].

#### 3.4.4. Prostacyclin Analogues

Safety and efficacy of prostacyclin analogues in PPHTN have been reported in some uncontrolled studies [55]. Careful monitoring is recommended for PPHTN patients receiving prostacyclin, as some developed thrombocytopenia and/or splenomegaly during treatment. In addition to intravenous epoprostenol, both intravenous and subcutaneous treprostinil, as well as inhaled iloprost, demonstrated positive short-term effects in PPHTN patients [56].

#### 3.4.5. Combination Therapy

In a recent study of 637 patients with PPHTN, Savale et al. [10] demonstrated that oral combination therapy with ERA and PDE-5i induced a significant reduction in PVR (−64%) compared to monotherapy with ERA (−40%) or PDE-5i (−37%). Furthermore, oral combination therapy showed significant mPAP reduction and CO and 6-MWD improvement. Analogous results were subsequently reported in PPHTN patients enrolled in the Japanese multicenter registry, where combination therapy was associated with mPAP, PVR, and CI improvements [57].

### 3.5. Trans Jugular Intrahepatic Portosystemic Shunt

There are limited data on trans jugular intrahepatic (TIPS) porto-systemic shunts to treat HPS patients. However, this procedure may worsen pulmonary circulation by increasing intrapulmonary vasodilation and worsening hypoxemia. Other side effects include hepatic decompensation and porto-systemic encephalopathy. Pulmonary artery embolization is a limited procedure and is used only in cases where there is notable vasodilation and exaggerated arterio-venous communication.

Although TIPS is considered a therapeutic option to reduce portal pressure, it is usually not indicated in patients with PPHTN, as it may increase portosystemic shunting and worsen pulmonary hypertension. TIPS is associated with hemodynamic changes, leading to increased right ventricular preload and afterload, which may negatively impact PPHTN patients’ survival. The presence of mPAP ≥ 35–44 mmHg (moderate PPHTN) is a possible contraindication to TIPS placement. Moreover, in the case of mPAP ≥ 45 mmHg (severe PHTN) and/or signs and symptoms of right-sided congestive heart failure, TIPS is contraindicated [58].

Differential diagnosis of hepato-pulmonary syndrome is reported in Table 4 (Ref. [59]).

## 4. HPS Outcome

After liver transplantation, patients should continue PAH-specific treatments, as liver transplantation is associated with hemodynamic instability in the postoperative period [6].

Regular follow-up, typically every 4–6 months, is recommended for patients with PPHTN undergoing liver transplantation. Transhepatic embolization (TTE) is considered the most appropriate follow-up approach, while routine right-sided endoscopic surgery (RHC) is generally unnecessary unless clinically indicated.

To date, no controlled study has determined the best strategy for discontinuing PAH-specific treatments after liver transplantation. Patients should be monitored in specialized centers to evaluate possible drug tapering based on hemodynamic status.

Although liver transplantation is not the ultimate treatment for PPHTN, hemodynamic normalization occurs in some patients within a year after transplantation. Current data on long-term outcomes in PPHTN are conflicting, with studies suggesting that 14–64% of patients can be weaned from PAH-specific therapy. The time of PAH-therapy discontinuation varies widely between studies [6]. Prognostic factors to predict resolution of PPHTN after liver transplantation have not been identified. Furthermore, cases of new-onset PAH after liver transplantation have been reported in patients with a previously normal hemodynamic profile or even with short-term hypersensitivity syndrome (HPS).

## 5. Future Directions

At present, PPHTN is a complex disease, and more research is needed to identify PAH-specific therapies. In this context, available data indicate that macitentan significantly lowers PVR. Patients with severe disease have a high mortality rate with or without LT, and long-term response to therapy is yet unknown. Moreover, it is unclear if hemodynamic parameters improve after LT, leading to survival benefit. Notably, the assessment of peri-operative risk by non-invasive evaluation of right ventricular function will favor hemodynamics management in mild liver disease and improve LT outcome [25]. Further points to be addressed include the following: (i) PPHTN treatment in MELD exception; (ii) risk of severe disease progression; (iii) high pre-operative risk of patients with mPAP > 35 mmHg based only on small retrospective case series; (iv) guidelines to start PAH treatment before LT and to continue PAH therapy post-LT; and (v) PPHTN national registries of liver transplanted patients [23].

Future HPS research lines suggest doing the following: (i) update MELD exception calculating hypoxemia level, PVR, RV function, and phenotype; (ii) define the lower cut off of the PRV definition; (iii) create prognostic risk scores associated with severe liver dysfunction and possible lung involvement; (iv) develop new multicenter drug studies [6].

## 6. Conclusions

PPHTN is a challenging disease, and further research is needed to clarify which types of PAH-specific therapies will be useful for patients with different disease severities. Moreover, the risk of progression and prognostic predictor factors are unknown. Lastly, pre-operative risk stratification of patients prior to liver transplant remains undefined. HPS has a severe outcome characterized by worsening hypoxemia and increasing vasodilation. Without liver transplant, HPS patients are destined for a quick death. However, only 80% of patients improve oxygenation and reduce arterio-venous shunts after liver transplantation. In a few cases, several complications occur, such as the development of relapsing HPS or severe post-liver transplant hypoxemia or even relapsing porto-pulmonary hypertension. Pathophysiology and treatment of these complex diseases require further research to improve the quality of patients’ care.

## Figures and Tables

**Table 1:** Porto-pulmonary hypertension and hepato-pulmonary syndrome definition.

Porto-Pulmonary Hypertension	Hepato-Pulmonary Syndrome
Group 1 PAH clinical classification	Liver disease (usually cirrhosis with portal hypertension)
PAH in patients with portal hypertension	Positive contrast-enhanced transthoracic echocardiography
Elevated mean pulmonary arterial pressure (mPAP)	Hypoxemia: alveolar-arterial gradient (A-a O_2_) ≥15 mmHg (or ≥20 if >65 years) Hypoxemia: O_2_ arterial partial pressure (PaO_2_) <70 mmHg
Increased pulmonary vascular resistance (PVR)	
Right-sided heart failure	Intrapulmonary vascular dilatations and/or shunting
High morbidity and mortality	

**Table 2:** Child-Turcotte-Pugh classification of chronic liver disease.

Child-Turcotte-Pugh Classification			
Score	1	2	3
Ascites	Absent	Moderate	Massive
Encephalopathy	Absent	Mild	Severe
Bilirubin (mg/dL)	<2	2–3	>3
Albumin (g/dL)	>3.5	2.8–3.5	<2.8
Prothrombin time (%)	>70	40–70	<40

Low (class A) 5–6 points, intermediate (class B) 7–9 points, and poor (class C) risk 10–15.

**Table 3:** Clinical trials reporting survival after liver transplantation in porto-pulmonary hypertension.

Authors	No. of Patients	Survival (%)		
		1 yr	3 yrs	5 yrs
Sadd et al.	24	87	87	87
Reymond et al.	23	83	83	--
Rajaram et al.	13	69	--	--
Ashfaq et al.	11	91	--	67
Cartin-Ceba et al.	50	72	63	60
Salgia et al.	78	85	81	--
Verma et al.	28	63	59	54
DuBrock et al.	103	86	–	--
Savale et al.	35	80	77	77
Savale et al.	63	92	83	81

Adapted from DuBrock HM (Ref [25]).

**Table 4:** Differential diagnosis and diagnostic procedures of hepato-pulmonary syndrome.

Differential Diagnosis	Diagnostic Procedures
Porto-pulmonary Hypertension	Contrast-enhanced echocardiography transesophageal echocardiogram
Atelectasis	Chest computed tomography
Recurrent pulmonary emboli	Pulmonary angiography
Atrial septal defect	Transesophageal echocardiogram
Arteriovenous malformations	Pulmonary angiography
Post-pneumonectomy	Echocardiography
Chronic cardiopulmonary disease	Chest computed tomography Pulmonary function tests
COPD	Pulmonary function tests Echocardiography
Pneumonitis	Chest X-ray
Hepatic hydrothorax	Total body computed tomography Magnetic resonance imaging
Ascites	Abdominal ultrasonogram

## Data Availability

Not applicable.
